# Distinct functions of wild-type and R273H mutant Δ133p53α differentially regulate glioblastoma aggressiveness and therapy-induced senescence

**DOI:** 10.21203/rs.3.rs-3370608/v1

**Published:** 2023-11-02

**Authors:** Curtis Harris, Sebastien Joruiz, Natalia Von Muhlinen, Izumi Horikawa, Mark Gilbert

**Affiliations:** NCI NIH; Center for Cancer Research, National Cancer Institute; Center for Cancer Research, National Cancer Institute; NCI; Center for Cancer Research, National Cancer Institute

## Abstract

Mutations effects on p53 isoforms’ activities remain largely unknown, although they are mutated in 92% of *TP53* mutant cancers. Therefore, exploring the effect of mutations on p53 isoforms activities is a critical, albeit unexplored area in the p53 field. In this article, we report for the first time a mutant Δ133p53α-specific pathway which increases IL4I1 and IDO1 expression and activates AHR, a tumor-promoting mechanism. Accordingly, mutant Δ133p53α R273H increases glioblastoma cancer cells proliferation and invasion while the WT does not. Furthermore, while WT Δ133p53α reduces apoptosis to promote DNA repair, the mutant also reduces apoptosis but fails to maintain genomic stability.Furthermore, both WT and mutant Δ133p53α reduce cellular senescence in a senescence inducer-dependent manner (temozolomide or radiation) because they regulate different senescence-associated target genes. Hence, WT Δ133p53α rescues temozolomide-induced but not radiation-induced senescence, while mutant Δ133p53α R273H rescues radiation-induced but not temozolomide-induced senescence. Lastly, using TCGA data, we determined that IL4I1, IDO1 and AHR are significantly higher in GBMs compared to LGGs. IL4I1 expression is increased in mutant *TP53* LGGs and GBMs, although only significantly in LGG. Importantly, high expression of all three genes in LGG and IL4I1 in GBM is significantly associated with poorer patients’ survival. These data show that, compared to WT Δ133p53α, R273H mutation reorientates its activities toward carcinogenesis and activates the oncogenic IL4I1/IDO1/AHR pathway, a potential prognostic marker and therapeutic target in GBM by combining drugs specifically modulating Δ133p53α expression and IDO1/Il4I1/AHR inhibitors.

## Introduction

*TP53*, the most frequently mutated gene in human cancers^1^, naturally expresses at least 12 different p53 isoforms, which share a central domain^2^. Therefore, in 92% of *TP53* mutant cancers, all p53 isoforms are mutated^3^. The effects of mutations on p53α activities, are well studied and include loss- and gain-of-functions^4^, but their effect on p53 isoforms activities remain unknown. Several clinical studies demonstrated that the prognosis accuracy of cancer patients could be improved by combining *TP53* mutation status and p53 isoforms expression^2,5–7^. Therefore, exploring the effect of mutations on p53 isoforms activities is a critical, albeit unexplored area in the p53 field.

Δ133p53α, one of the best characterized isoforms, is a negative regulator of senescence, particularly in normal cells^8–11^. Δ133p53α is also involved in DNA double-strand break repair and pluripotent stem-cells regulation where it prevents genomic instability^12^. Importantly, wildtype (WT) Δ133p53α is non-oncogenic and non-mutagenic in normal human cells^12^.

Glioblastoma (GBM) are the most aggressive brain tumors with only 6.8% five-year relative survival^13^. Current treatments, including temozolomide chemotherapy and radiations, induce cellular senescence through a p53-dependent mechanism^14,15^. The progression from low-grade astrocytoma to glioblastoma is accompanied by *TP53* mutations^16^. Since Δ133p53α prevents cellular senescence, studying the impact of its mutation in glioblastoma is relevant to determine if it affects the progression and response to treatment of GBM cells and mutant *TP53* cancers in general, whether it may be used as a prognostic marker, and represent a potential therapeutic target.

Here, we identified a new mutant Δ133p53α-specific pathway in which it increases IDO1 (indoleamine-2,3-dioxygenase 1) and IL4I1 (interleukin-4 induced 1) expression and activate AHR (aryl hydrocarbon receptor), a tumor promoting mechanism. We confirmed that mutant Δ133p53α R273H increases glioblastoma cells proliferation and invasion and prevents apoptosis while failing to maintain DNA stability. In addition, it also affects the response to treatment as WT and mutant Δ133p53α respond differently to temozolomide and radiation treatments.

## Material and methods

### Cell Culture and treatments

Wild-type *TP53* (U87-MG, A172) and *TP53*-null (LNZ-308) cells were obtained from ATCC. Mutant *TP53* cells (SF-268 and SNB-19) were obtained from NCI-Frederick cancer DCTD tumor/cell line repository. Cells were sequenced by CD Genomics^17^ to confirm their classification as GBM according to the 2021 World Health Organization’s classification of tumors of the central nervous system^18^ and validate their *TP53* status. Cells were cultured at 37°C, 5% CO2 in DMEM (Gibco^®^, Invitrogen) with 10% FBS, 1% L-Glutamine and 1% penicillin/streptomycin (Invitrogen).

When specified, cells were exposed to 50μM temozolomide (TMZ, Sigma-Aldrich) for 5 days or irradiated with 10 Grays X-rays (X-Rad 320 biologic irradiator, Precision X-ray) and analyzed 5 days after treatment.

### Lentiviral vectors transduction

Lentiviral particles production is described in supplementary methods. 1mL DMEM containing FLAG-Δ133p53α Wild-Type, FLAG-Δ133p53α R273H or control-RFP viral particles was added on cells for 24h. Cells were selected with 1μg/ml Blasticidin (Sigma-Aldrich).

### Western Blot

Cells were lysed in RIPA (Cell Signaling Technology) and quantified using Bradford assay (Biorad). Western blot procedure is detailed in supplementary methods. ECL (Amersham) reagent was applied to detect bands on Biorad imager. Antibodies used are listed in Supplementary table 1 and full-size images in Supplementary Fig. 5. MAP4^8^ (Moravian Biotechnologies), SAPU^19^, KJC12^20^.

### Transcriptome and Analysis

mRNAs were extracted using Qiagen Rneasy-Plus kit according to manufacturer’s instructions. mRNA sequencing (40M reads, paired-end 150 bp) was performed on NovaSeq6000 (Illumina) by the CCR sequencing facility (Frederick, MD, USA). Library preparation and reads alignment details are described in supplementary methods. Differential Expression Analysis (DEG) and Gene Set Enrichment Analysis (GSEA) were performed using the NIH Integrated Data Analysis Platform^21^ (NIDAP). Cutoff value used was Adjpval < 0.01, |logFC|>1. GEO accession number GSE240377.

### Quantitative Real-Time PCR (qRT-PCR)

Reverse-transcription and quantitative analysis were performed as previously described^22^. Primers used (all from ThermoFisher) are listed in Supplementary table 2. Expression level was analyzed with the ΔΔCt method and normalized to GAPDH.

### Immunofluorescence staining

Immunofluorescence was performed as previously described^23^. Slides were mounted with Vectashield^®^ Antifade mounting medium with DAPI (Vector laboratories) and imaged using Zeiss-780 confocal microscope. Antibodies used are listed in Supplementary table 1.

### Senescence-associated (SA)-β-gal Assay

(SA)-β-Galactosidase Staining Kit (Cell Signaling Technology, #9860S) was used following manufacturer’s protocol.

### IL-6 ELISA

Human IL-6 ELISA kit (Sigma-Aldrich) was used following manufacturer’s instructions.

### Transfections

siRNAs (10nM final concentration) were transfected using Lipofectamine RNAiMAX (Invitrogen) following manufacturer’s reverse-transfection protocol. si133 (5′-GGAGGUGCUUACACAUGUU-3′) and control (12,935 – 100) came from Invitrogen.

WT and mutant p53α or pcDNA3 control plasmids (0.5μg) were transfected using TurboFect (Thermofisher Scientific) following manufacturer’s reverse-transfection protocol.

### Cell confluence and annexin-V live cell imaging

Growth medium was supplemented with Incucyte^®^ Annexin-V Dye (Sartorius) at 1/400 dilution. Phase (confluency) and green fluorescence (Annexin-V) pictures were taken every four hours by the Incucyte-S3^®^ system (10x magnification).

### Sulforhodamine-B (SRB) staining

Cells were fixed with ice-cold 100% methanol for 15 minutes and washed five times with water. Cells were incubated for 30min with SRB solution (0.4% SRB, 1% acetic acid, and deionized water) and washed five times with 1% acetic acid. SRB staining was solubilized using 10mM unbuffered Tris and absorbance read at 570nm.

### Transwell assay

Cells were serum-starved for 48h before being seeded in serum-free medium in the top chamber of Corning^®^ Transwell^®^ 8.0μm pore polycarbonate membrane cell culture inserts (Sigma-Aldrich). The bottom chamber contained 10% FBS medium. After 16h, non-migrated and migrated cells were collected and counted.

### Statistical analysis

Data are presented as mean and standard deviation with comparisons made using a two-sided, unpaired Student’s t-test, of at least three independent experiments. Differences were considered significant at a value of * p ≤ 0.05, ** p ≤ 0.01, and *** p ≤ 0.001 or NS (not significant).

### TCGA RNA-sequencing analysis

IL4I1, IDO1 and AHR (FPKM) expression and *TP53* and IDH mutation data from 669 primary glioma patients (516 low-grade glioma and 153 glioblastoma) from The Cancer Genome Atlas (TCGA) database were downloaded from cBioPortal^24^. Associated clinical information was obtained from Genomics Data Commons. IL4I1, IDO1 and AHR expression levels were stratified by tumor type, IDH mutation and *TP53* mutation status. GraphPad Prism was used to generate Kaplan-Meier overall-survival analysis and log-rank test to compare overall-survival between groups with high (greater than the median) and low (lower than the median) IL4I1, IDO1 or AHR expression. Survival analysis was stratified by *TP53* mutation status.

## Results

### Mutant Δ133p53α R273H induces IL4I1 and IDO1 expression and activates AHR

To study whether Δ133p53α mutation impacts its activities, we selected the R273H mutation since this is the most frequent mutation in GBM (13.5% and 11.8%) and the first or second most frequent mutation in cancers (6.4% and 5.9%) according to the COSMIC and *TP53* databases^3,25^, respectively. Since Δ133p53α knock-down is not possible without also knocking-down Δ133p53β/γ and Δ160p53α/β/γ, we decided to stably overexpress WT Δ133p53α in WT *TP53* GBM cells (U87 and A172), and mutant Δ133p53α R273H in R273H mutant *TP53* GBM cells (SF268 and SNB19). We confirmed that both WT and mutant Δ133p53α are similarly overexpressed in nearly all cells using western blot (Fig, 1A) and immunofluorescence staining (Supplementary Fig. 1).

**Figure 1:**
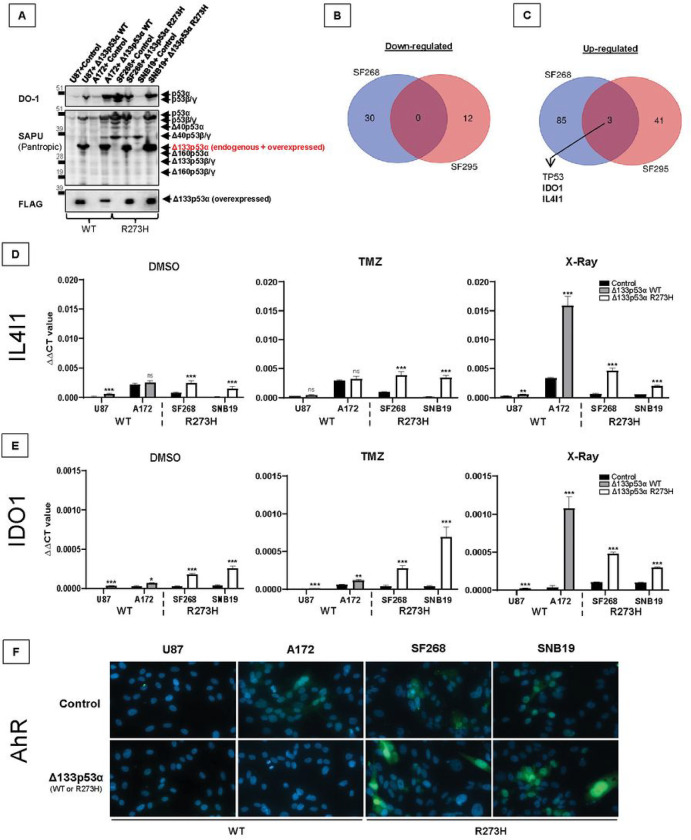
Mutant Δ133p53α R273H induces the IDO1/IL4I1/AHR pathway. **A)** Western blot of the WT or mutant FLAG-Δ133p53α and endogenous p53 isoforms using DO-1 (full-length p53 isoforms) SAPU (p53 pantropic) or anti-FLAG antibodies. n=3. **B and C)** Venn diagrams representing the down-regulated and up-regulated genes in SF268 and SNB19 following mutant Δ133p53α R273H overexpression. *TP53* is logically found up-regulated since we overexpressed the Δ133p53α isoform. **D and E)**IDO1 and IL4I1 mRNA expression measured by Taqman in cells treated with DMSO (control), TMZ (50μM for 5 days), or X-rays (10Gy). ΔΔCt values were normalized to GAPDH. n=3. **F)** Immunofluorescence staining (40x magnification) of AHR (Green) and nuclear stain DAPI (blue). n=3. AHR nuclear translocation is induced by mutant Δ133p53α R273H.

We treated Δ133p53α-expressing cells with temozolomide (TMZ) or X-rays and performed mRNA sequencing to determine the pathways and functions impacted by Δ133p53α mutation (Supplementary Fig. 1B-I). In WT cells, we could not identify common target genes up- or down-regulated by WT Δ133p53α overexpression. Similarly, no mutual down-regulated genes could be identified in mutant cells when comparing control versus mutant Δ133p53α R273H-overexpressing cells (Fig. 1B).

Nevertheless, we identified that, compared to control, mutant Δ133p53α R273H upregulates two target genes, IDO1 and IL4I1, shared between SF268 and SNB19 cells (Fig. 1C). Using qRT-PCR, we verified that IDO1 and IL4I1 are specifically up-regulated by mutant Δ133p53α R273H overexpression (Fig. 1D–E) while their expression was not or minimally increased by WT Δ133p53α. Of note, these two genes were highly increased by WT Δ133p53α in A172 cells, but not in U87 cells, and in response to radiations specifically, although these cell line- and treatment-specific changes are of unknown origin. To confirm that endogenous mutant Δ133p53α R273H also regulate IDO1 and IL4I1, we knocked-down the Δ133p53α-encoding transcript^2^ (Genbank NM_001126115) along with or without overexpression, which resulted in IDO1 and IL4I1 down-regulation (Supplementary Fig. 1J-L).

IDO1 and IL4I1 are involved in a common pathway where they induce AHR activation and nuclear translocation^26,27^. We confirmed that AHR expression is higher and more nuclear in mutant versus WT cells. Furthermore, upon mutant but not WT Δ133p53α overexpression, AHR is induced and translocated to the nucleus (Fig. 1F). Not all mutant Δ133p53α R273H-overexpressing cells are AHR positive, which might be explained by mutant Δ133p53α R273H heterogenous expression within cell population (Supplementary Fig. 1A). Furthermore, the IL4I1/IDO1/AHR axis promotes tumor progression and aggressiveness^26–30^ which is consistent with our Gene Set Enrichment Analysis (GSEA) indicating that mutant Δ133p53α R273H overexpression correlates with increased inflammatory response and epithelial-to-mesenchymal transition (EMT) (Supplementary Fig. 1M). These results indicate that mutant Δ133p53α R273H may have acquired oncogenic activities.

### Mutant Δ133p53α R273H promotes cell growth and invasion

**Figure 2:**
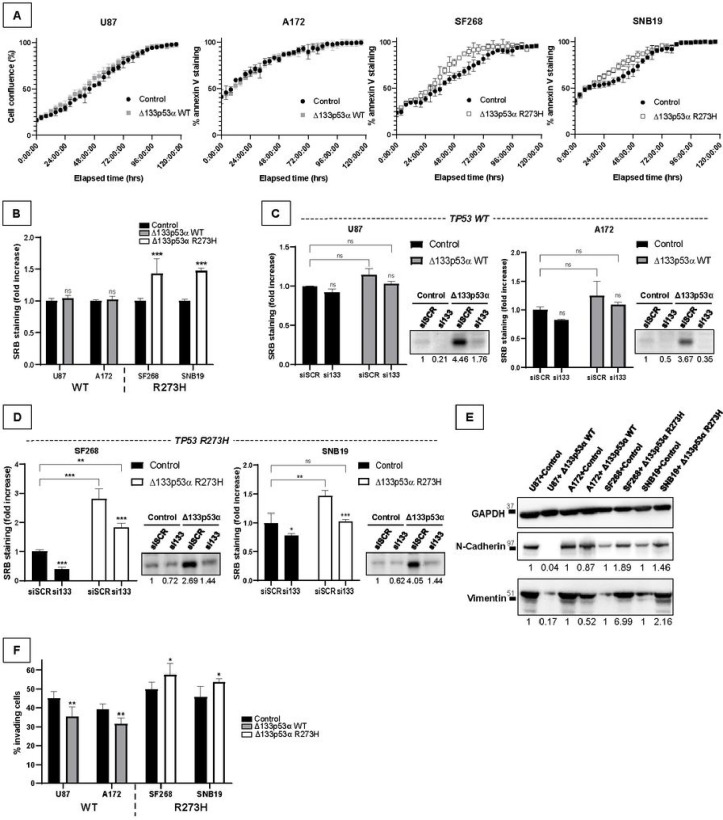
Mutant Δ133p53α R273H increases cell growth and cellular invasion. **A)** Cells were imaged every 4h in incucyte^®^ over 5 days and the percentage of confluence was measured. n=4. **B)** Cells were seeded at low density and left growing for 6 days before SRB staining was performed to determine cell growth. n=8. **C and D)** Cells were seeded and reverse transfected with siScr (control) or si133. After 6 days, cells were either used for western blot to assess the efficiency of Δ133p53 knock-down with MAP4 antibody or to determine cell growth by SRB. n=4. **E)** Protein expression of N-cadherin and Vimentin by Western blot. GAPDH was used as a loading control. n=3. **F)** Transwell assay was used to determine the percentage of invading cells 16h after seeding. n=5.

Previous studies of IL4I1/IDO1/AHR axis and our GSEA data both suggest that mutant Δ133p53α R273H may promote cell proliferation^26,27,30^. Therefore, we measured cell confluence over five days using the Incucyte^®^ (Fig. 2A). WT Δ133p53α-overexpressing cells grew similarly to control cells, while mutant Δ133p53α R273H expression minimally increased cell proliferation. This was reproducible in p53-null LNZ308 cells (Supplementary Fig. 2A-B), suggesting that mutant Δ133p53α R273H may promote cell growth in a p53α-independent manner, potentially via the IL4I1/IDO1/AHR axis. Using sulforhodamine-B (SRB) staining, we confirmed that WT Δ133p53α did not affect the cells’ growth rate, while mutant Δ133p53α increased proliferation by approximately forty-five percent (Fig. 2B). Furthermore, WT Δ133p53α knock-down did not affect cell growth while mutant Δ133p53α R273H depletion decreased cell proliferation, including in the Δ133p53α R273H-overexpressing cells (Fig. 2C–D).

Both the IL4I1/IDO1/AHR literature and our GSEA indicate that mutant Δ133p53α may increase EMT and cellular invasion^26,27,29^. Therefore, we analyzed EMT markers expression and found that mutant Δ133p53α R273H induced both N-cadherin and vimentin expression, while WT Δ133p53α reduced it (Fig. 2E). Using transwell assay, we observed that WT Δ133p53α overexpression slightly reduced cell invasion through the membrane (Fig. 2F). However, mutant Δ133p53α R273H overexpression significantly increased the percentage of invading cells, further suggesting mutant Δ133p53α oncogenic potential. In p53-null LNZ308 cells, WT Δ133p53α did not reduce cell invasion, suggesting a p53-mediated function, while mutant Δ133p53α R273H still increased invasion, indicating a p53-independent mechanism, potentially through the IL4I1/IDO1/AHR axis (Supplementary Fig. 2C).

### Mutant Δ133p53α R273H lost DNA repair but retains anti-apoptotic functions

**Figure 3:**
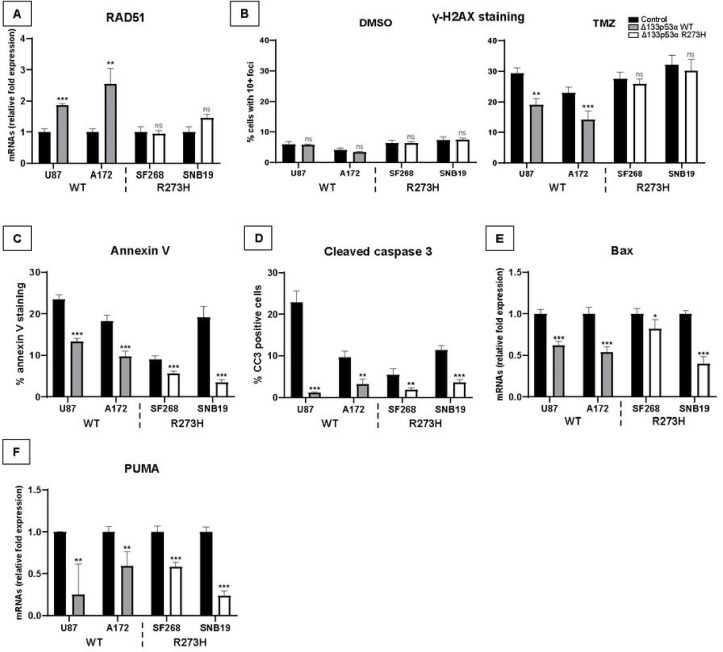
Mutant Δ133p53α R273H fails to promote DNA repair but retains anti-apoptotic functions. **A)** Fold change of RAD51 mRNA expression measured by Taqman. n=3. **B)** γ-H2AX staining was performed on cells treated with DMSO (control) or TMZ (50μM for 5 days). The percentage of cells with at least 10 foci was determined (corresponding images in Supplementary Fig. 3B). n=3 **C)** Cells were grown in the presence of annexin-V dye in Incucyte^®^ and the percentage of annexin-V staining was determined after 80 hours. n=4. **C)** Quantification of the percentage of cells positive for Cleaved Caspases 3 by immunofluorescence (corresponding images in Supplementary Fig. 3D). n=3. **E and F)** Bax and PUMA mRNA expression was measured by Taqman. n=3.

Δ133p53α promotes DNA repair through DNA repair genes upregulation, including LIG4, RAD51, and RAD52^31,32^. Hence, Δ133p53α maintains DNA integrity in induced pluripotent stem-cells and enhances DNA repair in prematurely aged cells through p53α inhibition and E2F1 activation^33–35^. Using qRT-PCR, we verified that WT Δ133p53α overexpression increases RAD51 expression while mutant Δ133p53α R273H did not affect it (Fig. 3A). Using Δ133 isoforms siRNA knock-down, we confirmed that, contrarily to the WT protein, endogenous mutant Δ133p53α R273H does not contribute to RAD51 expression (Supplementary Fig. 3A), suggesting that it may have lost its DNA repair function.

Next, we performed γ-H2AX staining to quantify DNA double-strand breaks. Under control conditions, the low DNA-damaged cells percentage did not allow to detect significant differences (Fig. 3B, Supplementary Fig. 3B). TMZ, an alkylating agent, induces DNA damage and represses E2F1-associated DNA repair pathway^14^, the pathway Δ133p53α activates to promote DNA repair^33–35^. Following TMZ treatment, WT Δ133p53α-overexpressing cells harbored a reduced number of γ-H2AX foci (Fig. 3B, Supplementary Fig. 3B). Mutant Δ133p53α R273H, however, did not prevent γ-H2AX foci formation, confirming that the mutation impaired Δ133p53α DNA repair activity.

To favor DNA repair, WT Δ133p53α represses p53α-mediated apoptosis ^8,36^. Since the IL4I1/IDO1/AHR axis prevents apoptosis^30,37^, mutant Δ133p53α R273H may retain its anti-apoptotic functions. Performing annexin-V and cleaved caspase-3 staining, we found that both WT and mutant Δ133p53α reduced spontaneous apoptosis (Figs. 3C–D, Supplementary Fig. 3D). Furthermore, we also performed annexin-V staining in p53-null LNZ-308 cells and neither WT nor mutant Δ133p53α reduced annexin-V staining (Supplementary Fig. 3C). This is consistent with WT Δ133p53α repressing p53α-mediated apoptosis and indicates that Δ133p53α R273H may also reduce apoptosis in a p53-dependent manner.

To investigate how Δ133p53α represses apoptosis, we quantified Bax and PUMA expression in Δ133p53α-expressing cells. This showed that both genes were reduced by WT and mutant Δ133p53α overexpression (Figs. 3E and 3F). Consistently, Bax and PUMA were up-regulated upon endogenous Δ133p53 isoforms knock-down (Supplementary Fig. 3E-F), confirming the negative correlation between Δ133p53α (WT or R273H) and Bax/PUMA expression. Altogether, these results demonstrate that Δ133p53α R273H retains its anti-apoptotic function, while failing to maintain DNA integrity.

### R273H mutation alters Δ133p53α regulation of cellular senescence, particularly in response to treatment

**Figure 4:**
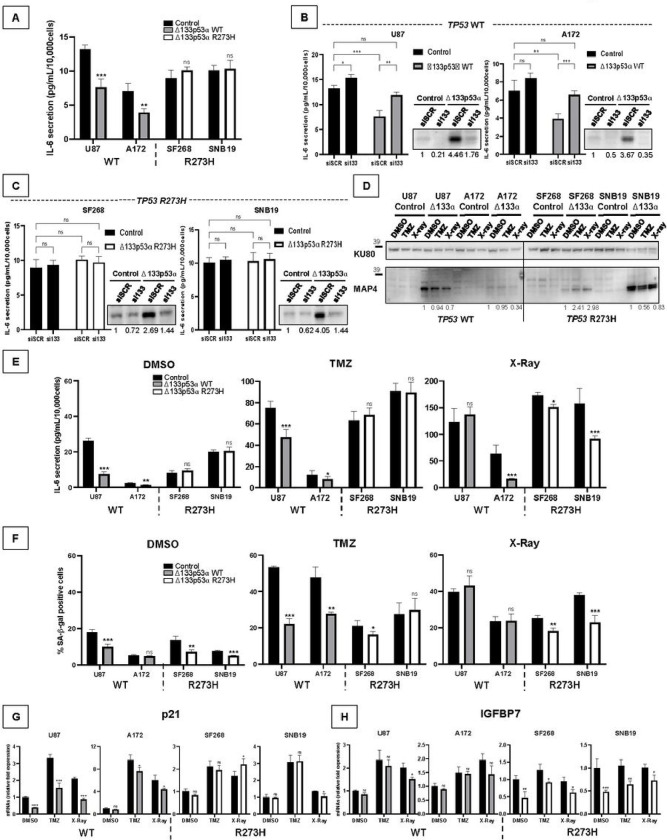
R273H mutation alters Δ133p53α regulation of cellular senescence, particularly in response to treatment. **A)** IL-6 secreted in growth media was measured by ELISA after 5 days. IL-6 secretion was normalized to the number of cells. n=4. **B and C)** Cells were seeded, and reverse transfected with siScr (control) or si133. After 5 days, cells were counted, and proteins were extracted for western blot to assess the efficiency of Δ133p53 knock-down with MAP4 antibody (same images as Fig. 2C and 2D as experiments were made at the same time), and IL-6 secreted in growth media was measured by ELISA. n=4. **D to F)** Cells were treated with DMSO (control), TMZ (50μM for 5 days), or X-rays (10Gy). Western blot of Δ133p53 isoforms (MAP4 antibody) was performed and KU80 was used as a loading control. n=3. (D). IL-6 secretion was measured by ELISA and normalized to cell number. n=3. (E). The percentage of senescent cells was determined by Senescence-associated β-galactosidase staining. n=3 (F). **G and H)** Fold change of p21 and IGFBP7 mRNA expression measured by Taqman in cells treated with DMSO (control), TMZ (50μM for 5 days), or X-rays (10Gy). n=3.

Our transcriptome analysis suggested that mutant Δ133p53α R273H induces inflammation via IL-6/JAK/STAT3, IL-2/STAT5, and TNFα/NFκB pathways (Supplementary Fig. 1M) while WT Δ133p53α reduces cellular senescence through inhibition of the senescence-associated secretory phenotype (SASP), including many inflammation factors^8–10,23,33^. To investigate whether R273H mutation alters Δ133p53α regulation of senescence and SASP, we first measured IL-6 secretion. Consistent with previous findings^8–10,23,33^, WT Δ133p53α overexpression reduced IL-6 secretion (Fig. 4A). In contrast, mutant Δ133p53α R273H did not affect IL-6 secretion. Furthermore, following Δ133p53 isoforms knock-down, IL-6 secretion was rescued in the WT cells but was unchanged in the mutant cells (Fig. 4B–C).

TMZ and radiation treatments are known cellular senescence inducers through a p53-dependent mechanism^14,15^. Both treatments reduced WT Δ133p53α expression, with radiation causing the greatest effect (Fig. 4D). While differences appeared between the mutant cells, in SNB19, mutant Δ133p53α R273H was reduced by TMZ, but not by radiation, suggesting that mutant and WT cells may differently respond to treatments. Both treatments induced IL-6 secretion in WT and mutant cells (Fig. 4E). In U87 cells WT Δ133p53α overexpression lowered IL-6 secretion in response to TMZ only. In A172 cells, IL-6 secretion was also decreased upon radiations which could be linked to the IDO1 and IL4I1 increase observed in response to radiations only in A172 cells (Figs. 1D–E). This indicates that WT Δ133p53α consistently counteracts TMZ-induced, but not radiation-induced IL-6 secretion. In contrast, mutant Δ133p53α R273H only decreased radiation-induced IL-6 secretion (Fig. 4E).

In the p53-null LNZ-308 cells, neither WT nor mutant Δ133p53α overexpression changed IL-6 secretion (Supplementary Fig. 4A), probably because Δ133p53α represses IL-6 secretion mainly through interaction with canonical p53α^8–10,23,33^. To test this hypothesis, we transiently overexpressed p53α in our GBM cells. Both WT and mutant p53α increased IL-6 secretion, consistent with previous reports that p53α R273H up-regulates pro-inflammatory pathways^38^. When co-overexpressed, only WT Δ133p53α counteracted WT p53α effect, while mutant Δ133p53α R273H could not prevent p53α R273H induction of IL-6 secretion. These results confirm WT Δ133p53α p53α-dependent mechanism while mutant Δ133p53α lost this function (Supplementary Fig. 4B-C).

Next, we asked whether IL-6 secretion correlated with cellular senescence. Both WT and mutant Δ133p53α reduced cellular senescence under control conditions (Fig. 4F). Interestingly, WT Δ133p53α rescued TMZ-induced senescence, but not radiation-induced senescence, while mutant Δ133p53α R273H rescued radiation-induced senescence but not TMZ-induced senescence, which is consistent with the IL-6 secretion above (Fig. 4E).

Although both can reduce senescence, WT and mutant Δ133p53α respond differently to treatment suggesting that they act through different mechanisms. We quantified senescence-associated target genes and found that WT Δ133p53α reduces p21 expression while mutant Δ133p53α R273H does not change it (Fig. 4G). In contrast, mutant Δ133p53α R273H reduced IGFBP7 expression whereas WT Δ133p53α had no effect (Fig. 4H). Using siRNA knock-down, we confirmed that p21 expression was upregulated by WT Δ133p53α knock-down only, while IGFBP7 was up-regulated upon mutant Δ133p53α knock-down only (Supplementary Fig. 4D-E). IGFBP7 downregulation was shown as a mechanism to escape p53α-induced senescence^39,40^. This indicates a switch from cell cycle arrest to senescence-associated IGF pathway induced by Δ133p53α mutation. The different target-gene selection may explain why both WT and mutant Δ133p53α can reduce cellular senescence in the absence of treatment, and their different response to treatment.

### IL4I1 expression is upregulated in GBM and is associated with poorer survival in GBM and LGG cohorts

**Figure 5:**
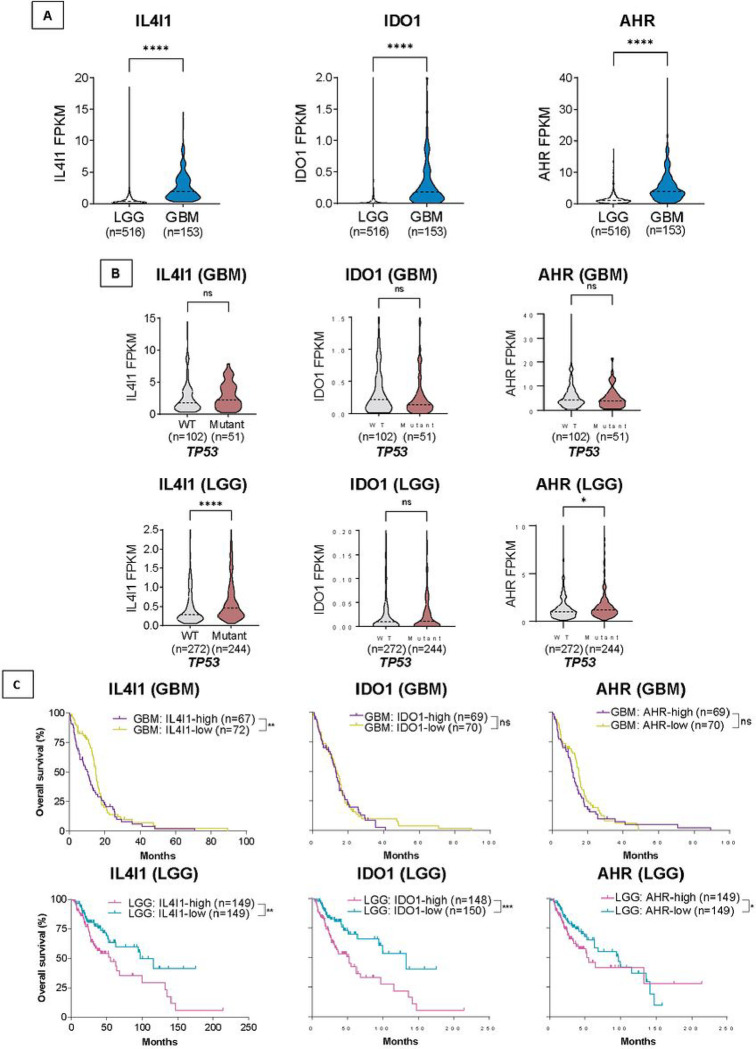
Expression of IL4I1, IDO1 and AHR is upregulated in glioblastoma patients compared to low-grade glioma patients and high levels of IL4I1 are significantly associated with *TP53*mutant tumors and poorer survival. Expression of IL4I1, IDO1 and AHR in **(A)** glioblastoma (GBM, n=153) compared to low-grade glioma patients (LGG, n=516), and **(B)** stratified by tumors harboring wildtype (n=140) or mutant (n=51) *TP53*. (C) Kaplan-Meier curves depicting the overall survival of GBM or LGG patients expressing high or low levels of IL4I1. P-values are indicated in graphs as * p ≤ 0.05, ** p ≤ 0.01, and *** p ≤ 0.001 or ns (not significant, p > 0.05). Mann-Whitney test was used to analyze statistical significance of A and B. Log-rank Mantel-Cox was used to determine significance of C.

We analyzed the TCGA database to examine IL4I1, IDO1, and AHR expression and their contribution to glioblastoma and low-grade glioma (LGG) patients’ clinical outcome. We also downloaded isocitrate dehydrogenase (IDH) mutational data to classify glioblastoma cases based on the 2021 World Health Organization directions establishing glioblastoma as grade IV astrocytomas with WT IDH^18^. We found that IL4I1, IDO1, and AHR expression are significantly higher in GBMs versus LGGs (Fig. 5A). We next stratified GBM and LGG patients by *TP53* mutation status, combining all mutants since there are less than 10 *TP53* R273H cases within the cohorts. We found that IL4I1 expression is higher in mutant versus WT *TP53* GBM and LGG tumors, though statistically significant only in LGG patients (Fig. 5B). In contrast, IDO1 expression is unchanged between mutant and WT *TP53* GBM or LGG cases, while AHR expression levels are significantly higher in mutant versus WT *TP53* LGG cases only. Unfortunately, the lack of isoform data within the TCGA database prevents the analysis of the association between IL4I1, IDO1 and AHR expression and Δ133p53α expression levels and mutation status.

We next stratified GBM and LGG patients by high (higher than median) or low (lower than median) IL4I1, IDO1 or AHR expression. Notably, higher IL4I1 expression is associated with shorter survival of GBM and LGG patients (Fig. 5C). Nevertheless, higher IDO1 and AHR expression were associated with poorer survival of LGG patients only. These results are consistent with our *in vitro* data showing that IL4I1 and IDO1 upregulation and AHR nuclear translocation by mutant Δ133p53α lead to higher tumor cell migration and invasion. Overall, these findings underscore IL4I1 prognostic significance in GBM and LGG and strengthen the potential therapeutic value of targeting IL4I1 expression in these tumors.

## Discussion

Here, we identified a novel, mutant-specific Δ133p53α/IDO1/IL4I1/AHR axis. Interestingly, these genes promote tumor progression and aggressiveness, including reduced apoptosis, increased proliferation, and resistance to treatment^26–30,37,41–43^. Additionally, IL4I1, IDO1, and AHR reduce T-cell proliferation and recruit suppressive immune cells^44,45^. Whether their induction by mutant Δ133p53α R273H leads to tumor microenvironment immunity alterations will require further investigations. Nevertheless, we report for the first time IL4I1, IDO1, and AHR are all three increased in GBM compared to LGG. Furthermore, we show that IL4I1, IDO1, or AHR high expression correlate with poorer survival of LGG patients, and that high IL4I1 expression also correlates with poorer outcome in GBM patients, confirming the clinical relevance of this pathway in these cancers.

**Figure 6:**
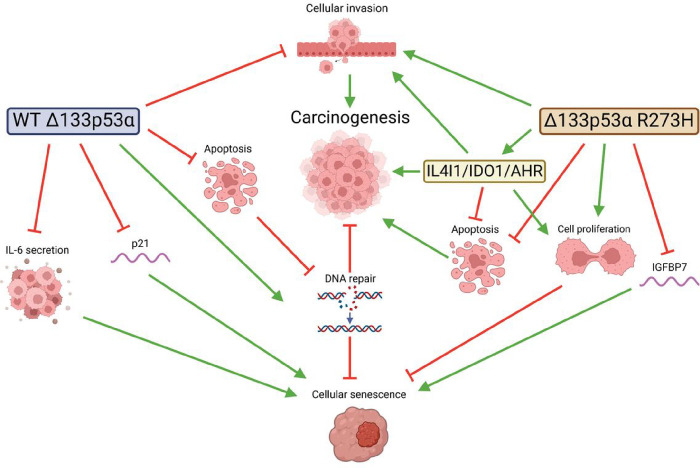
Model - Mutant Δ133p53α R273H mutation reorientates its activities towards carcinogenesis. Created with BioRender.com

We also determined that mutant Δ133p53α R273H increases glioblastoma cells proliferation, DNA instability, and invasion while reducing apoptosis, suggesting that it has gained oncogenic function (Fig. 6). Importantly, the increase in proliferation and invasion are mutant p53α-independent, indicating that these activities are actively carried by mutant Δ133p53α R273H. These activities may be attributed to IL4I1, IDO1 increased expression, and AHR activation by mutant Δ133p53α R273H, since they increase cancer cells proliferation, motility, and invasion^26,27,41–43^. IL4I1/IDO1/AHR depletion in mutant Δ133p53α-expressing cells will be required to determine the extent of their contribution to mutant Δ133p53α R273H biological activities. However, some activities (apoptosis and senescence) were mediated in a p53-dependent manner, suggesting that several mechanisms may co-exist. Importantly, WT Δ133p53α inhibits apoptosis to favor DNA repair pathways but does not prevent p53α-dependent apoptosis in severely damaged cells^31,32^. However, mutant Δ133p53α R273H has lost DNA repair capabilities and, therefore, cannot maintain genetic stability while blocking damaged cells elimination, further demonstrating that it acquired oncogenic functions. Lastly, we determined that Δ133p53α may have an impact on patient’s response to treatment. Indeed, both WT and mutant Δ133p53α reduce cellular senescence and expression of senescence-associated genes, but in an inducer of senescence-dependent manner. Hence, WT Δ133p53α, but not the mutant, reduces TMZ-induced senescence while mutant Δ133p53α R273H, but not the WT, prevents radiation-induced senescence.

Altogether, these findings provide novel mechanistic insights into mutant Δ133p53α R273H activities and demonstrate that it is an active contributor to glioblastoma carcinogenesis and response to therapeutic treatment. Hence, a mutant *TP53* tumor expressing mutant Δ133p53α may be more aggressive and not respond to treatment the same way as a mutant *TP53* tumor not expressing mutant Δ133p53α. Our results suggest future prognosis opportunities by combining *TP53* mutation status and isoforms expression. By discovering the link between *TP53* mutation and IL4I1/IDO1/AHR pathway, we show strong evidence of Δ133p53α R273H clinical relevance in mutant *TP53* glioblastoma development and aggressiveness, and as a potential therapeutic target and biomarker. Since IL4I1 and IDO1 are specifically induced by mutant Δ133p53α, targeting them or AHR in mutant *TP53* tumors may offer new clinical opportunities^46,47^. Several IDO1 small-molecule inhibitors are in clinical trials for advanced melanoma^48,49^. While the first phase III trial was not conclusive^50^, specifically targeting mutant *TP53* tumors or combining it with mutant Δ133p53α-targeting drugs may improve effectiveness. Similarly, several AHR antagonists exist, and piperazine-2,3-dione derivatives were suggested as selective IL4I1 inhibitors^51^. Interestingly, IL4I1 is secreted and found in serum^52^ where it promotes a tumor-prone microenvironment, increasing the concentration of metabolites in the patient’s biological fluids, including malignant gliomas patients’ cerebrospinal fluid^53–55^. Therefore, this may be an opportunity to detect mutant Δ133p53α R273H-induced expression of IL4I1 in mutant *TP53* tumors in a non-invasive and easier way.

## Data Availability

Data generated for this manuscript will be made available upon reasonable request to the corresponding author. The mRNA sequencing results have been deposited to the Gene Expression Omnibus (GEO) and can be found under the accession number GSE240377.
